# Wind in November, Q Fever in December

**DOI:** 10.3201/eid1007.030724

**Published:** 2004-07

**Authors:** Hervé Tissot-Dupont, Marie-Antoinette Amadei, Meyer Nezri, Didier Raoult

**Affiliations:** * Unité des Rickettsies, Faculté de Médecine, Marseille, France;; † Centre Hospitalier Général, Martigues, France

**Keywords:** Q fever, *Coxiella burnetii*, epidemiology, human transmission, wind

## Abstract

Q fever, a worldwide zoonosis caused by *Coxiella burnetii*, can be transmitted from animal reservoirs to humans by the inhalation of infected aerosols. We investigated the epidemiology of Q fever in the Bouches-du-Rhone district of southern France, particularly the role of wind and rainfall in *C. burnetii* transmission. During the winter of 1998 to 1999, an unexpected number of cases were diagnosed in the area. This statistically higher incidence was associated with an increased frequency of the mistral 1 month before onset of disease, i.e., shortly after the main lambing season. These data confirm that wind plays a role in *C. burnetii* transmission, a factor that can be monitored but not prevented. Further studies are needed to identify and confirm preventable individual behavioral risk factors for Q fever.

## Abstract 2

An investigation in southern France confirms the role of wind in *Coxiella burnetii* transmission

Q fever is a worldwide zoonosis caused by *Coxiella burnetii*, an obligate intracellular bacteria which lives in the phagolysosomes of the host cell. The main characteristic of Q fever is its clinical polymorphism. Acute cases, which are symptomatic in less than 50%, generally manifest as an insolated fever or a flulike syndrome that may be accompanied by granulomatous hepatitis, pneumonia, or meningoencephalitis (*1*). Cases with febrile eruptions, myocarditis, and pericarditis have also been reported (*2*); the various clinical manifestations may depend on host factors (*2*). In chronic Q fever, endocarditis is the primary sign (*3*), although osteomyelitis, infections of vascular grafts or aneurisms (*4*), and pregnancy complications (*5*) have also been reported. Thus, a serologic confirmation is required for the diagnosis of Q fever.

Throughout the world, the most common reservoirs of *C. burnetii* are cattle, sheep, and goats (*6*); the bacterium is found in urine, feces, milk, and birth products of infected animals (*7*). Also, infected cats (*8*), rabbits (*9*), and dogs (*10*) can transmit *C. burnetii* to people. Human infections mainly occur after persons inhale contaminated aerosols and, rarely, after they ingest unpasteurized milk or cheese.

The role of wind in aerosol transmission has been suggested since the 1950s (*11*). Two large outbreaks of Q fever have been studied extensively and have provided additional information about the disease’s epidemiology. In a British study (*12*), Q fever developed in persons who were exposed to contaminated straw, manure, and dust introduced by the vehicles that traveled along the road where these persons lived. In a Swiss study (*13*), Q fever also developed in 415 persons who lived on a valley road along which sheep were herded to mountain pastures. Table 1 summarizes the other main outbreaks reported over the last 20 years.

**Table 1:** Primary Q fever outbreaks reported over the last 20 years

Source	Year	Country	No. of cases	Reference
Sheep	1981	USA	81	14
	1982	UK	14	15
	1983	Switzerland	415	13
	1993	Italy	58	16
	1996	Germany	45	17
	1996	Germany	18	18
	1996	France	204	19;20
Cattle	1982	USA	25	15
	1996	Poland	25	21
Goats	1992	France	40	22
	1998	Slovakia	113	23
	2000	Canada	62	24
Cats	1984	Canada	13	25
	1988	Canada	12	8
	1989	USA	15	26
Rabbits	1986	Canada	4	9
Pigeons	2000	France	4	27
Dogs	1996	Canada	3	10

In a survey carried out from 1995 to 1997 (*28*), the study area (40 km northwest of Marseille) was shown to have an incidence of Q fever 5.4 times higher than that of the area of Marseille. This hyperendemicity could be due to wind blowing through an extensive sheep-rearing area before reaching the study area. The main peak of Q fever cases occurs in April and May in the disease-hyperendemic area, 1 month after the second lambing season, which takes place when the strongest winds blow. At the time of the main lambing, in October and November, the wind is infrequent, leading to a small number of Q fever cases.

During the winter of 1998 to 1999, an unexpected number of cases were diagnosed in this area. Our study attempted to confirm our previous hypotheses by correlating this unusual winter peak of infections with unusual meteorologic events.

## Materials and Methods

### Serologic Diagnosis

The serologic diagnosis of Q fever was performed at the National Reference Center by using the immunofluorescence reference technique as previously described (*29*). The titers of immunoglobulin (Ig) G, IgM, and IgA against phases I and II of *C. burnetii* were determined. A case of evolving Q fever (acute or chronic) was diagnosed when the phase II IgG titer was >200 and the phase II IgM titer was >50. A diagnosis of chronic Q fever was made when the phase I IgG titer was >800 (29).

### Demographic, Geographic, and Meteorologic Data

**Figure 1:**
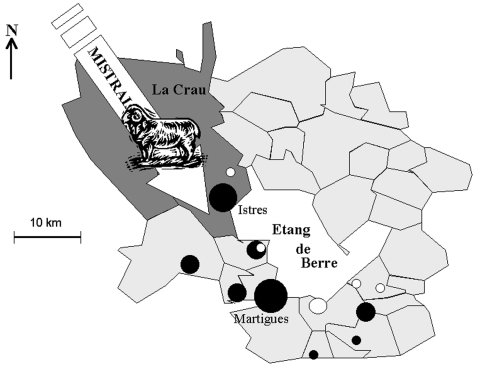
Etang de Berre area of France, showing the location of "La Crau" (sheep-breeding area), and the direction of the mistral wind. The black dots represent the human Q fever cases (places of residence). The white dots represent the 7 cases which occurred in December 1998 to January 1999.

Marseille is a city located in southern France with 1 million inhabitants. About 40 km northwest of Marseille is a large, natural, saltwater lake called "Etang de Berre" (Figure 1). Northwest of Etang de Berre is a 600-km^2^, semidesert plain region called "La Crau," which is the only steppe in Western Europe. La Crau is limited by the Alpilles Mountains (north), the Mediterranean Sea (south), the Etang de Berre (west), and the Rhône River (east). The irrigated northern area (humid Crau) is devoted to hay cultivation (120 km^2^), whereas the stony and dry southern part is devoted to sheep grazing.

Data on sheep breeding were obtained from the "Chambre d’Agriculture" in Aix en Provence. These data included the approximate number of sheep and the features of sheep breeding in the area.

Meteorologic data were obtained from the Meteo France weather station at Istres in the form of cumulative wind speed and direction compass cards from January 1996 to December 1999 and monthly compass cards and data sheets for November and December between 1996 and 1999. Wind speed and direction are measured every 3 hours, providing about 240 data points per month. The tables provided by Meteo France show the wind direction and speed in three meter per second (m/s) speed ranges of 2–4, 5–8, and >8. We considered only the mistral, which is the most common and strongest wind and blows for several consecutive days, in sunny and dry conditions. It mostly originates from between the west-northwest and the north and blows through La Crau before reaching the study area. Winds that occur in other local climatic conditions, such as rainfall and high humidity, come primarily come from other directions, mainly the east and south, and blow for short periods only. These winds were not considered in our study. Monthly cumulative precipitation data between 1996 and 1999 in Istres, and daily precipitation for October, November, and December, 1998 and 1999, were also obtained from Meteo France (www.meteo.fr).

Using the serologic criteria described above, *Coxiella burnetii*–positive patients were identified from the database of the National Reference Center between 1996 and 1999. The eastern part of La Crau, along the Etang de Berre, was considered the study area. All patients had conditions diagnosed when they were inpatients or outpatients at the general hospital in Martigues or in private laboratories in the cities of Martigues, Fos-sur-Mer, Istres, Sausset-les-Pins, and Châteauneuf-les-Martigues (outpatients who were sent to the laboratory by their general practitioner). Medical practices in this area send all serum specimens for Q fever diagnosis to the National Reference Center or, if they test serum specimens by immunofluorescence with phase II antigen provided by the National Reference Center, send positive serum specimens to the center for confirmation. All serologic diagnoses of Q fever are therefore ultimately made at the National Reference Center with the reference serologic technique. Therefore, we may assume that all diagnosed cases are included in our database. For each patient with a diagnosis of Q fever, a questionnaire was filled out, which provided administrative, epidemiologic, clinical, and biologic data. Patient data were included in the study only if the patient’s place of residence (as recorded in the hospital or laboratory files) was in the study area.

### Statistical Analyses

All data were managed by using EpiInfo 6 (Centers for Disease Control and Prevention, Atlanta, GA). Pearson’s chi square or two-tailed Fisher exact test was used to compare frequencies of qualitative data. A difference was considered significant when p < 0.05.

## Results

### Q Fever Incidence and Monthly Distribution

**Figure 2:**
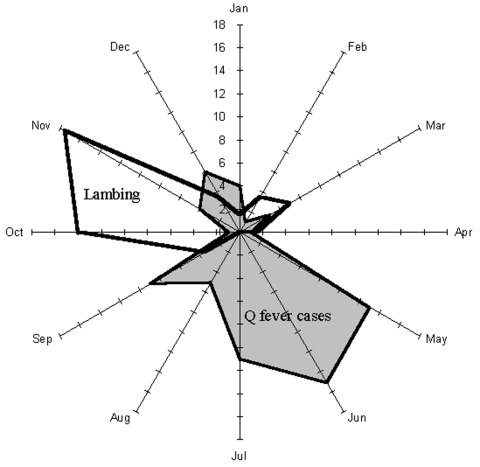
Seasonal variations of Q fever incidence and sheep births in the Etang de Berre area of France: cumulative cases for 1996–1999.

**Figure 3:**
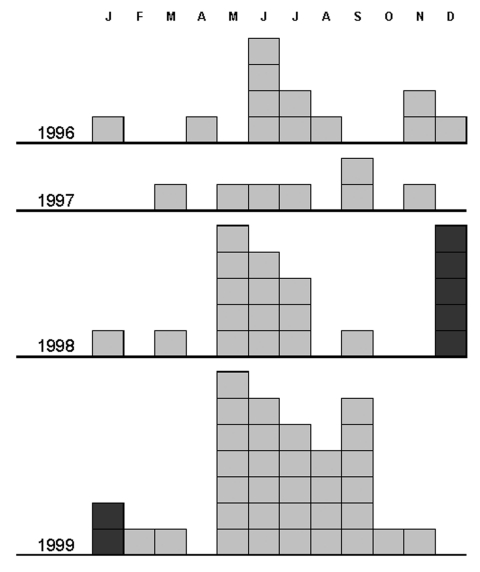
Monthly distribution of Q fever cases in the Etang de Berre area of France: comparison between the years 1996–1999, showing an unusual peak in December 1998 and January 1999.

Between 1996 and 1999, acute Q fever was diagnosed in 73 patients in the study area. Figure 2 shows the cumulated monthly distribution of these cases (based on date of onset of symptoms) and indicates the usual main peak in incidence of infection in late spring (May–June). When this distribution was considered by year (Figure 3), an unusual peak was observed in December 1998 (5 cases) and January 1999 (2 cases). The proportion of cases occurring in December 1998, in relation to the total number of cases in 1998 (5/20), was significantly higher than the proportion of cases occurring in December in the other study years (1/53) (p < 0.006, Fisher exact test).

The seven patients whose diagnosis was made in December 1998 and January 1999 were interviewed: they were six men and one woman, with a mean age of 34 years. Two case-patients had cardiac valve disorders. Four of them reported living in rural areas. One of them lived in La Crau (Entressen), and six lived on the opposite (southeastern) shore of Etang de Berre (Châteauneuf-les-Martigues, Saint Mitre, Marignane, and Gignac) (Figure 1). None reported an occupational exposure. Two case-patients reported a usual exposure to sheep; one had been exposed to parturient sheep. Two persons owned a cat, and four owned a dog. None of these pets was a newborn or had given birth recently. No patients reported consuming unpasteurized milk or cheese.

### Sheep Breeding in La Crau

Sheep have been bred in La Crau for centuries. The main lambing season (80% of births) is in October and November (Figure 2) and takes place either indoors or outdoors. In the latter case, birth products (mainly placentas) are left on the ground where they desiccate and can be a source of fomite spread of *C. burnetii*. Between October and February, sheep are allowed to graze hay in the northern humid part of La Crau. A second lambing season (20% of births) occurs in March. Between March and June, sheep graze in the dry southern part of La Crau. In June, the sheep are moved by trucks to their summer pastures in the Alps so sheep are generally absent from La Crau until September.

### Meteorologic Factors: Wind Frequency and Rainfall

During the 4 years of the study, the mistral represented 44.9% of all winds >2 m/s, 59.4% of winds >5 m/s, and 80% of winds >8 m/s, which confirms the importance of the mistral in terms of frequency of occurrence and speed. To explain the unusual peak of Q fever cases in December 1998 and January 1999, we studied the wind frequencies 1 month before, i.e., in November and December. Table 2 shows the percentage of northwest wind for the three defined speed ranges, by year; p refers to the comparison between the year under consideration and 1998. In each speed range, the frequency of the mistral was higher in November and December 1998 than in any other year: for the speed range >2 m/s, it was 60.9% in November to December 1998, whereas it was 41.3, 40.5, and 52.4 in November to December 1996, 1997, and 1999, respectively. This unusual frequency was even more notable for the strongest winds (which are more consistent with the mistral), representing 93.5% of the wind in November to December 1998 but only 63.1%, 47.7%, and 85% in November to December 1996, 1997, and 1999, respectively. All these differences were statistically significant, except those between 1998 and 1999 in the speed range >5 m/s.

**Table 2:** Northwest wind (mistral) in November and December 1996 to 1999, expressed as the percentage of measures for three wind speed ranges

Years	Northwest wind >2 m/s (%)	p^a^	Northwest wind >5 m/s (%)	p^a^	Northwest wind >8 m/s (%)	p^a^
Nov–Dec 1996	41.3	<10^–7^	47.3	<10^–7^	63.1	<10^–6^
Nov–Dec 1997	40.5	<10^–7^	50.0	<10^–7^	47.7	<10^–7^
Nov–Dec 1998	60.9		76.9		93.5	
Nov–Dec 1999	52.4	0.01	72.3	0.21 (NS)^b^	85.0	0.045

^a^p values refer to the comparison between the considered year and 1998. ^b^NS, not significant.

**Figure 4:**
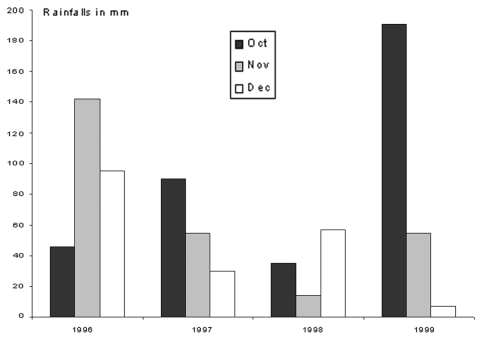
Rainfall (in mm) in October, November, and December 1996 to 1999 in Istres.

**Figure 5:**
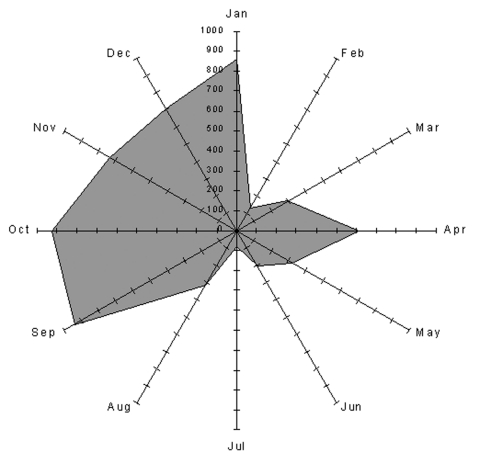
Mean monthly rainfall in Istres (1995–1999).

In terms of precipitation (Figure 4), October and November 1999 were much more rainy (190.6 mm and 54.4 mm of rainfall, respectively) than October and November 1998 (34.8 mm and 14.2 mm, respectively). The rainfall amount of December 1999 (7.2 mm) was lower than that of December 1998 (56.8 mm). Exceptionally high amounts of precipitation (156.6 mm) were noted in October 1999. The global precipitation of December 1998, primarily depends on that of December 31 (43.4 mm of 56.8 mm). Figure 5 shows the mean monthly rainfall from 1995 to 1999. The main lambing in autumn occurs during the period of the most rainfall, whereas the spring secondary lambing occurs during a dry period.

## Discussion

In our previous study, carried out in the same area, we showed a geographic and statistical relationship between the sheep densities, the incidence of Q fever, and the strong, local wind known as the mistral, which blows from the northwest (*28*). Although *C. burnetii* transmission is multifactorial, we speculated that the high incidence of Q fever in the study area was related to aerosol spread of organisms because the mistral blows through the local steppe where 70,000 sheep are bred (Figure 1). This study was designed to confirm this hypothesis and find an explanation for the unusual peak of Q fever cases that occurred during the winter of 1998 to 1999. We found that no changes occurred in medical practice or demographics of the region during the study period, and the Chambre of Agriculture and the veterinary services reported no unusual events in the sheep flocks in the region. Under these conditions, we have shown the following: 1) the incidence of Q fever was statistically higher in the period from December 1998 through January 1999 than in the same period in other years; 2) that the speed of the mistral, especially, was significantly higher in November to December 1998 than in other years. Our study has then shown that the increased frequency of the mistral blowing through La Crau after the main lambing season was associated with an unusual peak of Q fever cases in the study area 1 month later. However, Table 2 also shows that the mistral blew with increased frequency during the 1999 winter (although significantly less so than in 1998), without a significant increase in the incidence of Q fever cases. We suspect this might be related to variations in the conditions of *C. burnetii* transmission which are known to be strongly multifactorial. One of these factors seems to be rainfall, since the fall of 1998 was drier than that of other years, particularly that of 1999, when the mistral was also stronger than in the other 2 years. Moreover, we have shown that the main fall lambing takes place at a time when the mistral is unfrequent, and the environment is wet. On the contrary, the secondary spring lambing occurs at a time when a strong mistral blows, at a dry season, which enhances the aerosols.

The role of wind in the aerosol transmission of *C. burnetii* has been suggested since the 1950s (*11*). The wind probably played a role in Q fever cases which occurred in northern Kent (United Kingdom) in people living and working near a fertilizer factory which received offal from abattoirs in Kent and Sussex (*11*). A small outbreak occurred in a kindergarten in France caused by aerosol transmission from cattle manure infected with *C. burnetii* applied to nearby pastures (*30*). An epidemic in people exposed to packing straw has been described in eastern Kent (United Kingdom) (*11*), and another epidemic occurred in workers exposed to dust from maize grain used as animal food (*31*). The role of wind has also been suspected in the infection of cotton (in fields or assembly areas) from neighboring sheep and cattle pastures (*32*). More recently, the role of wind was assessed in a large outbreak in Birmingham (United Kingdom) in 1989. A case-control study (26 case-patients and 52 matched controls) produced no evidence that direct contact with animals or animal products had caused the outbreak. The epidemic curve suggested a point source exposure in the week beginning April 10. The home addresses of case-patients were clustered in a rectangle-shaped area, 11 miles (18.3 km) north/south by 4 miles (6.7 km) east/west, and attack rates became lower toward the north. Directly south of this area was a region containing farms where outdoor lambing and calving took place, a potent source of *C. burnetii* spores. A retrospective computerized analysis showed that the geographic distribution of cases was associated with a source in this area (p < 0.00001). On 11 April, unusual southerly gales of up to 78 mph (130 km/h) were recorded. The probable cause of the outbreak was windborne spread of *C. burnetii* spores from farmland to more settled areas (*33*). The role of wind, however, was excluded in a study conducted from 1996 to 2000 in French Guiana (*34*). In the study of a 1987 outbreak in a Somerset (United Kingdom) secondary school, the high prevalence of unexplained infections was suspected of being related to the spread of organisms, either windborne or in straw or manure (*35*). In Germany, 40 outbreaks of human Q fever were documented from 1947 to 1999 (*36*). Sheep were implicated in the transmission in at least 24 outbreaks. Dry weather or wind blowing from areas where sheep were located to inhabited areas likely contributed to at least 14 episodes.

Evidence is now accumulating that wind is an epidemiologic factor in Q fever outbreaks occurring near sheep-rearing areas. It is a factor that can only be monitored and not prevented. Some preventive measures could be of interest in terms of public health: serologic testing and vaccinating sheep, indoor lambing, and appropriate disposal of placentas and litter. However, the feasibility of such measures in France in general and particularly in La Crau is low: Q fever is not a veterinarian-reportable disease, so testing (in cases of abortion) is carried out on request and must be paid by the farmer. An effective phase I animal vaccine is not yet available in France. Although indoor lambing is not possible in such an extensive breeding area, due to the lack of sheepfolds, recommendations have been made on proper disposal of placentas (they should not be left on the ground where they dry up, but properly collected and incinerated). This is possible only when lambing occurs in the presence of farmers.

Many other personal and behavioral factors may be involved that would be preventable. Further studies are needed to identify and confirm such preventable risk factors.
